# Dichotomous thinking about food as an understudied subclinical disordered eating cognition

**DOI:** 10.1186/s40337-025-01388-7

**Published:** 2025-10-15

**Authors:** Jordan A. Levinson, Jordan E. Parker, Jeffrey M. Hunger, A. Janet Tomiyama

**Affiliations:** 1 Nutrition and Food Sciences Department, University of Vermont, 109 Carrigan Drive, 256 MLS Carrigan Wing, Burlington, VT 05405 USA; 2 Department of Psychology, University of California Los Angeles, 1285 Franz Hall, Box 951563, Los Angeles, CA 90095 USA; 3 Department of Psychology, Miami University, 90 N Patterson Avenue, Oxford, OH 45056 USA

**Keywords:** Dichotomous thinking about food, Disordered eating, Eating disorder

## Abstract

Dichotomous thinking about food involves sorting foods into strict categories such as “good” and “bad” or “healthy” and “unhealthy,” and is an understudied cognition in the context of disordered eating. Although this way of thinking is an established symptom in orthorexia nervosa, there is a dearth of research on dichotomous thinking about food and its correlates and consequences. The purpose of this paper is to discuss the lack of research on dichotomous thinking about food, as well as to understand previously unknown associations between dichotomous thinking about food and other subclinical disordered eating behaviors and cognitions. In a racially diverse sample of 630 women ages 18–44, dichotomous thinking about food was positively and significantly (at *p* < .01) associated with body dissatisfaction, binge eating, cognitive restraint, restriction, excessive exercise, purging, drive for thinness, and anti-fat attitudes. Results suggest that dichotomous thinking about food warrants further inclusion in research on eating disorder pathology. Future studies should examine the prevalence of dichotomous thinking across ages, gender and racial/ethnic groups, consequences of this cognitive pattern, and whether dichotomous thinking about food precedes eating disorder diagnoses.

In American culture, individuals tend to sort food into strict, opposing categories such as “healthy” vs.”unhealthy,” or “good” vs. “bad” [18], engaging in dichotomous thinking about food (DTAF). These food-related descriptors often stem from their perceived healthfulness and influence on potential weight changes [17]. Indeed, “healthy” foods are typically described as “natural” or “nutritious” [8] and are thought to be associated with desirable weight loss [4, 5]. In contrast, “unhealthy” foods are routinely labeled “unnatural” and “processed” and consumption of these foods is associated with undesirable weight gain and guilt [5]. This categorization has proliferated in mainstream food marketing, capitalizing on the societal value of thinness, often using language to indicate that food is morally good (e.g., “guilt-free” snacks) [9].

Cognitive-behavioral theories of eating disorders identify rigid food rules or strict black and white thinking about food as feature of clinical eating disorders [26]. Such strict categorization of food is also a common feature of orthorexia nervosa [16], a subclinical eating disorder characterized by “a pathological fixation on eating proper food” [1, 21]. Individuals with orthorexia believe that eating or avoiding certain foods will promote wellness to the point of mental preoccupation with the goal of optimum health [21]. Many subclinical disordered eating cognitions (i.e., body dissatisfaction, drive for thinness, purging) are precursors to the development of clinical eating disorders [15, 22, 23]. These cognitions have been extensively studied in myriad populations [12, 13]. To the extent that DTAF affects food choices aimed at achieving thinness and avoiding fatness, as well as shaping how individuals feel about their bodies, it is evident that this eating-related cognition may be a subclinical symptom or precursor to eating disorder development. However, dichotomous thinking about food–and its role as a subclinical disordered eating cognition–has been understudied. Given that eating disorders have one of the highest mortality rates among all psychological disorders [24], it is imperative to both understand their contributors and prevent their development.

Although an understanding of food-related dichotomous thinking is clearly warranted, most research in this domain has examined *general* dichotomous thinking, defined as “the propensity to think of things in terms of binary opposition” [18]. This research shows that global dichotomous thinking is in fact positively associated with global disordered eating scores [11], dieting, and dietary restraint [25] in both community and clinical samples of women. In the few studies that focus on dichotomous thinking about food specifically, evidence shows that DTAF is significantly higher among dieters than non-dieters [19]. DTAF is also significantly and positively correlated with restrained eating [19], disinhibited eating, and overvaluation of weight and shape [14]. Given that behaviors such as dieting and dietary restraint are classified as clear risk factors for later eating disorder diagnosis [22], these aforementioned associations are cause for concern.

Although considerable research has addressed more common subclinical disordered eating cognitions such as drive for thinness or body dissatisfaction [12, 22], there is a dearth of work on dichotomous thinking about food. To this end, the aim of the current exploratory analysis is to test if dichotomous thinking about food is associated with an array of established subclinical disordered eating behaviors and cognitions.

## Method

### Participants

This study was approved by the UCLA Institutional Review Board. A non-clinical sample was recruited from the UCLA Psychology undergraduate participant pool and Prolific. Eligibility criteria included identifying as a woman or as non-binary, and being 18 years of age or older. Those who completed the study through the SONA system were rewarded 0.5 research participation credits towards a class of their choosing, and prolific participants were compensated $5.50 for completing the study. Data from 630 women or non-binary participants were included in the current analysis. Participants ranged from 18 to 45 years old, with an average age of about 20 years old, and were 30.6% Asian/Asian American, 21.4% white, 17.0% Latine, 15.4% Black, 9.8% more than one race, 4.3% Middle Eastern, and 0.5% Native American. Participants were also asked to report how they perceive their weight; 42% perceived themselves to be “about the right weight,” while about 44% perceived their weight as slightly overweight, overweight, or very overweight, and about 13% perceived their weight as slightly underweight, underweight, or very underweight.

### Measures

#### Disordered eating cognitions and behaviors

Disordered eating cognitions and behaviors over the past four weeks were measured using the Eating Pathology Symptoms Inventory (EPSI; [6]). Subscales included Body Dissatisfaction (7 items; α = 0.88), Binge Eating (8 items; α = 0.89), Cognitive Restraint (3 items; α = 0.80;), Excessive Exercise (5 items; α = 0.89), Restricting (6 items; α = 0.86), Purging (6 items; α = 0.89), and Negative Attitudes Toward Obesity (referred hereafter as Anti-Fat Bias, 5 items; α = 0.88). Response options ranged from “Never” to “Very Often” and items were scored from 0 to 4. Subscale values were created by summing items, with higher scores indicating more disordered eating cognitions and behaviors. Drive for thinness was measured using the Drive for Thinness subscale of the EDI-3. This subscale included seven items (α = 0.82) scored on a scale from 0 to 4 with response options ranging from “Never” to “Always.” Higher scores indicate a higher drive for thinness.

#### Dichotomous thinking about food (DTAF)

DTAF was measured using the Eating subscale of the Dichotomous Thinking in Eating Disorders Scale [2, 3]**.** This subscale includes four items such as “I think of food as either ‘good’ or ‘bad’,” and “When dieting, I view my eating as having been either good or bad” (α = 0.86; range = 0–16). Participants were asked to indicate how true each item was to them. Response options ranged from “Not at all true of me” to “Very true of me” and were scored from 1 to 4 with higher scores indicating more dichotomous thinking about food.

## Results

As expected, dichotomous thinking about food was significantly and positively correlated with all measured disordered eating cognitions and behaviors (all *p* < 0.01). Table 1 includes means and standard deviations for, and correlations between, all study variables.

**Table 1:** Variable descriptives and correlations among all study variables

	M(SD)	DTAF	BD	BE	COG	RESTR	EX	AFB	PURGE
DTAF	9.96 (3.51)	–	–	–	–	–	–	–	–
BD	14.80 (6.54)	0.55*	–	–	–	–	–	–	–
BE	12.69 (7.32)	0.47*	0.52*	–	–	–	–	–	–
COG	4.87 (3.24)	0.45*	0.44*	0.35*	–	–	–	–	–
RESTR	8.62 (5.74)	0.20*	0.24*	0.11*	0.21*	–	–	–	–
EX	6.74 (5.38)	0.27*	0.28*	0.30*	0.51*	0.17*	–	–	–
AFB	4.09 (4.27)	0.31*	0.26*	0.34*	0.34*	0.05	0.32*	–	–
PURGE	2.21 (4.20)	0.39*	0.38*	0.40*	0.45*	0.27*	0.40*	0.30*	–
DT	11.23 (6.91)	0.68*	0.67*	0.48*	0.60*	0.22*	0.34*	0.30*	0.50*

^*^p < .01

*DTAF* : dichotomous thinking about food; *BD*: body dissatisfaction; *BE*: binge eating; *COG*: cognitive restraint; *RESTR*: restriction; *EX*: excessive exercise; *AFB*: anti-fat bias; *PURGE*: purging; *DT*: drive for thinness

## Discussion

Previous research has found a relationship between global dichotomous thinking and higher global disordered eating and dieting [11, 19, 25]. More limited research has also established a relationship between dichotomous thinking about food in particular and more dieting, restrained eating [11, 19], overvaluation of weight and shape, and disinhibited eating [14]. However, the association between dichotomous thinking about food and a wider array of subclinical disordered eating cognitions and behaviors has not yet been explored. The current study found significant positive correlations between dichotomous thinking about food and a range of other disordered eating cognitions and behaviors in a diverse sample of women. Interestingly, DTAF was highly correlated with drive for thinness, but was only modestly associated with restriction. This may be because all items on the EPSI Restricting subscale ask about the amount of food eaten, whereas DTAF is focused on whether the content of food makes it acceptable or unacceptable to eat. Additionally, this finding may be due to participants labeling food dichotomously, but not physically restricting those foods. Indeed, self-reported attitudes and cognitions often modestly correlate with actual behavior, and multiple factors shape when and how our thoughts or attitudes translate into behavioral action [7]. Given that the observed correlations showed medium to strong associations for many measures, it seems likely that DTAF is closely related to the cognitive and behavioral states that characterize subclinical EDs and precede the onset of clinical diagnoses.

The positive relationship between DTAF and a range of disordered eating cognitions and behaviors suggests that further rigorous empirical study of this construct is warranted. It seems vital to establish whether DTAF should be considered a disordered eating cognition, and further, its role as a risk factor for the development of clinical eating disorders. Indeed, in clinical samples, dichotomous thinking about food appears to be common among individuals with orthorexia nervosa, a subthreshold eating disorder with serious consequences such as malnutrition [21]. Yet it remains to be established whether DTAF might also be a common precursor to other eating disorders, such as anorexia nervosa and bulimia nervosa.

## Limitations and future directions

As an exploratory study, the current investigation is not without its limitations. First, the Dichotomous Thinking about Food measure [2], was created to be used with dieters and includes dieting-specific items; however, individuals in this study were not required to be dieters to participate. Therefore, these items may not have captured a more general sense of DTAF. This analysis also relies on a self-report measure, which may result in subjectivity or inaccuracy in responses [20]. Additionally, our cross-sectional study and correlational analyses cannot rule out potentially causal or confounding factors (e.g., gender, experiences of weight stigma) that may explain the observed associations. To this end, the field would benefit from creating and validating a measure of dichotomous thinking about food aimed at the general population, and/or including a subscale measuring dichotomous thinking in larger measures of disordered eating such as the EPSI. Moreover, future research should test how this construct relates to subclinical disordered eating both within and across demographic factors such as race/ethnicity and gender.

Nonetheless, with an ever-present negative societal attitude towards fatness and foods associated with weight gain [10], understanding potential negative effects of dichotomous thinking about food is essential. As eating disorders remain prevalent and dangerous, affecting 28.8 million Americans in their lifetime [24], there is an increasingly pressing need to determine the cognitive and behavioral factors associated with these disorders.

## Data Availability

The datasets used and/or analyzed during the current study are available from the corresponding author on reasonable request.
